# DC-SIGN Induction in Alveolar Macrophages Defines Privileged Target Host Cells for Mycobacteria in Patients with Tuberculosis

**DOI:** 10.1371/journal.pmed.0020381

**Published:** 2005-11-15

**Authors:** Ludovic Tailleux, Nhan Pham-Thi, Anne Bergeron-Lafaurie, Jean-Louis Herrmann, Patricia Charles, Olivier Schwartz, Pierre Scheinmann, Philippe H Lagrange, Jacques de Blic, Abdellatif Tazi, Brigitte Gicquel, Olivier Neyrolles

**Affiliations:** 1Institut Pasteur, Unité de Génétique Mycobactérienne, Paris, France,; **2**Hôpital Necker-Enfants-Malades, AP-HP, Service de Pneumologie et d'Allergologie Pédiatrique, Paris, France,; **3**Hôpital Saint-Louis, AP-HP, Service de Pneumologie, Paris, France,; **4**Hôpital Saint-Louis, AP-HP, Service de Microbiologie, Paris, France,; **5**Institut Pasteur, Groupe Virus et Immunité, Paris, France,; **6**Centre National de la Recherche Scientifique, URA 2172, Paris, France; Oxford UniversityUnited Kingdom

## Abstract

**Background:**

Interplays between *Mycobacterium tuberculosis,* the etiological agent of tuberculosis (TB) in human and host professional phagocytes, namely macrophages (Mφs) and dendritic cells (DCs), are central to immune protection against TB and to TB pathogenesis. We and others have recently shown that the C-type lectin dendritic cell–specific intercellular adhesion molecule-3 grabbing nonintegrin (DC-SIGN; CD209) mediates important interactions between mycobacteria and human monocyte-derived DCs (MoDCs) in vitro.

**Methods and Findings:**

In order to explore the possible role of DC-SIGN in *M. tuberculosis* infection in vivo, we have analysed DC-SIGN expression in broncho-alveolar lavage (BAL) cells from patients with TB (*n* = 40) or with other non-mycobacterial lung pathologies, namely asthma (*n* = 14) and sarcoidosis (*n* = 11), as well as from control individuals (*n* = 9). We show that in patients with TB, up to 70% of alveolar Mφs express DC-SIGN. By contrast, the lectin is barely detected in alveolar Mφs from all other individuals. Flow cytometry, RT-PCR, and enzyme-linked immunosorbent assay analyses of BAL-derived fluids and cells indicated that *M. tuberculosis* infection induces DC-SIGN expression in alveolar Mφs by a mechanism that is independent of Toll-like receptor-4, interleukin (IL)-4, and IL-13. This mechanism most likely relies on the secretion of soluble host and/or mycobacterial factors that have yet to be identified, as both infected and uninfected bystander Mφs were found to express DC-SIGN in the presence of *M. tuberculosis*. Immunohistochemical examination of lung biopsy samples from patients with TB showed that the bacilli concentrate in pulmonary regions enriched in DC-SIGN-expressing alveolar Mφs in vivo. Ex vivo binding and inhibition of binding experiments further revealed that DC-SIGN–expressing alveolar Mφs constitute preferential target cells for *M. tuberculosis,* as compared to their DC-SIGN^−^ counterparts. In contrast with what has been reported previously in MoDCs in vitro, ex vivo DC-SIGN ligation by mycobacterial products failed to induce IL-10 secretion by alveolar Mφs, and IL-10 was not detected in BALs from patients with TB.

**Conclusion:**

Altogether, our results provide further evidence for an important role of DC-SIGN during TB in humans. DC-SIGN induction in alveolar Mφs may have important consequences on lung colonization by the tubercle bacillus, and on pulmonary inflammatory and immune responses in the infected host.

## Abstract 2

Alveolar macrophages from patients with TB express higher levels of DC-SIGN compared with controls. Ex-vivo infection induced DC-SIGN in infected and bystander cells, which made the latter more susceptible.

## Introduction

Interactions between *Mycobacterium tuberculosis,* the airborne agent of tuberculosis (TB) in human and host phagocytes, namely macrophages (Mφs) and dendritic cells (DCs), are central to anti-mycobacterial immunity and to TB pathogenesis [1,2]. In particular alveolar Mφs constitute a primary niche for the tubercle bacillus [3]. At the molecular level, interactions between the bacillus and host phagocytes rely on a variety of cellular receptors, among which the C-type lectin DC-specific intercellular adhesion molecule-3 grabbing nonintegrin (DC-SIGN; CD209) has recently received particular attention. Initially described as an HIV gp120 receptor [4,5], DC-SIGN has afterwards been shown to allow monocyte-derived DCs (MoDCs) to recognize a variety of microbes, including viruses, parasites, and bacteria [6]. A set of recent reports has established that DC-SIGN plays a key role in mycobacteria interactions with DCs, at least in vitro [7,8]. Indeed, DC-SIGN allows human MoDCs to recognize members of the *M. tuberculosis* complex through lipoarabinomannan (LAM), an abundant lipoglycan of the mycobacterial envelope [7,9], and through other molecules, such lipomannan, arabinomannan, and the 19 kDa antigen [8]; and DC-SIGN ligation by LAM partially inhibits the maturation of bacterial lipopolysaccharide (LPS)-stimulated MoDCs and potentiates the secretion of the anti-inflammatory cytokine interleukin (IL)-10 by these cells [8]. DC-SIGN interactions with mycobacteria or mycobacterial products may thus be of benefit for either the pathogen, by down-modulating DC functions, or for the host, by limiting tissue inflammation and immunopathology [11–13]. It is noteworthy that DC-SIGN is expressed by human pulmonary DCs [7,14], and mycobacteria-derived antigens have been detected in DC-SIGN^+^ DCs in lymph nodes from patients with TB, suggesting that interactions between the lectin and the bacillus may occur during the natural course of infection [7].

In order to further our understanding of the role of DC-SIGN in vivo, we have analysed the expression of the lectin in broncho-alveolar lavage (BAL) cells from patients with TB, as compared to cells from other patients.

## Methods

### Patients and Samples

Individuals studied (*n* = 74; mean age 17.6 ± 19.3 y; median age 10.5 y; 54% males) included patients with TB (*n* = 40), sarcoidosis (*n* = 11), or asthma (*n* = 14). Other individuals had alveolar proteinosis (*n* = 1), tracheal obstruction (*n* = 1), tracheal surgery (*n* = 1), inactive TB (*n* = 5), and lymphoma (*n* = 1). These individuals (*n* = 9) are considered as controls in the rest of the study. All individuals were HIV negative and non-smokers. None were receiving anti-mycobacterial therapy and/or corticosteroids at the time of biopsy, except for the treatment of asthma. TB was diagnosed by smear observation and/or bacterial culture and/or clinical symptoms. Asthma was defined as a history of recurrent wheezing episodes and daily use of asthma medication. The diagnosis of sarcoidosis was based on previously described criteria [16]. Inactive TB refers to patients who had a previous history of TB (which was successfully treated) without reactivation. BAL fluids were performed as previously described [17] for diagnostic purpose. Blood mononuclear cells from healthy volunteers (Etablissement Français du Sang, Paris, France) and from TB patients with smear observation were isolated by Ficoll-Paque (Pharmacia, Uppsala, Sweden) centrifugation. Only fluids in surplus were used in this study, according to institutional guidelines.

### Alveolar Mφs Isolation, Treatment, and Infection

BAL cells were washed and cultured in RPMI-1640 (Invitrogen, Carlsbad, California, United States) supplemented with 10% heat-inactivated foetal calf serum (FCS; Dutscher, Brumath, France) and 2 mM L-glutamine in Petri dishes for 1 h at 37 °C before removing nonadherent cells. When required, cells were treated with IL-4 (10 ng/ml, R&D Systems), tumor necrosis factor (TNF)-α (50 ng/ml; R&D Systems, Minneapolis, Minnesota, United States), *Escherichia coli*–derived LPS (100 ng/ml; Sigma, St. Louis, Missouri, United States), or a green fluorescent protein (GFP)-expressing strain of *M. tuberculosis* H37Rv (pEGFP plasmid was a kind gift from G. R. Stewart, University of Surrey, United Kingdom) at a multiplicity of infection (MOI) of one bacterium per cell.

### THP1 Cell

THP1 ( ATCC TIB-202) cells were transduced with a lentiviral DC-SIGN–encoding vector [18]. DC-SIGN–expressing cells were positively selected by flow cytometry–based cell sorting and cultivated, as THP1 cells, in RPMI-1640 supplemented with 10% heat-inactivated FCS and 2 mM L-glutamine.

### Flow Cytometry

Cells were treated and analysed as previously described [19]. The following monoclonal antibodies (mAbs) were used: anti-CD11b-PE, anti-CD11c-PE, anti-CD16-PE, anti-CD32-PE, anti-CD40-PE, anti-CD64-FITC, and anti-CD206-PE (all from Beckman Coulter, Allendale, New Jersey, United States); anti-CD1a-PE, anti-CD11b-APC, anti-CD14-PE, anti-CD83-PE, anti-CD86-PE, anti-CD123-PE-Cy5, and anti–human leukocyte antigen (HLA)-DR-PE (all from BD Biosciences, San Diego, California, United States); anti-CD209 (DC-SIGN)-FITC and anti-PE (clone 120507; R&D Systems); anti-TLR2-PE, anti-TLR4-PE, and anti-TLR9-PE (all from eBioscience, San Diego, California, United States); and anti-BDCA-1-APC, anti BDCA-2-PE, and anti-BDCA-3-PE (all from Miltenyi Biotech, Bergisch Gladbach, Germany). Isotype controls were all purchased from BD Biosciences. Fluorescence was analyzed using FACScalibur and CellQuest Pro software (BD Biosciences).

### Binding Experiments

Total BAL cells were pre-incubated for 30 min at 37 °C, according to a previously published procedure [20], in RPMI-1640 containing 10% FCS and eventually containing isotype controls, either a mix of PE-conjugated and unconjugated anti-DC-SIGN antibodies (clones 120507 and 1B10, a kind gift from A. Amara, Institut Pasteur, Paris, France) or a mix of APC-conjugated and unconjugated anti-CD11b antibodies (clones M1/70 [BD Biosciences] and 2LPM19c1 [Dako, Glostrup, Denmark]). All antibodies were used at 10 μg/ml. Pre-incubation at 37 °C in the presence of antibodies resulted in partial internalization of the corresponding receptors but did not decrease cell-associated fluorescence, because a mix of unlabelled and fluorescently labelled antibodies was used during this step (data not shown). Cells were then infected with GFP-expressing *M. tuberculosis* H37Rv, at a multiplicity of infection of five bacteria per cell, for 4 h at 4 °C to allow binding without phagocytosis. Cells were then washed in RPMI-1640 and further stained with PE-conjugated anti-DC-SIGN and APC-conjugated anti-CD11b antibodies for 30 min at 4 °C. Cells were then analyzed by flow cytometry. Binding to THP1 and THP1::DC-SIGN cells was realized as previously described for DC-SIGN–expressing HeLa cells [7]. Inhibition of binding was realized using anti–DC-SIGN antibodies at 10 μg/ml.

### Enzyme-Linked Immunosorbent Assay

BAL fluids were centrifuged for 15 min at 1000 × *g* and supernatants were concentrated three to eight times using Ultra-15 and YM-5 concentrators (Amicon; Millipore, Billerica, Massachusetts, United States). Concentrated BAL fluids were analyzed for IL-4, IL-10, and IL-13 using high-sensitivity enzyme-linked immunosorbent assay (ELISA) kits from R&D Systems. The minimal detectable dose of IL-4, IL-10, and IL-13 was 0.11 pg/ml, <0.5 pg/ml, and <32 pg/ml, respectively. For analysis of alveolar Mφ supernatants, Mφs were cultured in Petri dishes for 1 h before removing non-adherent cells. Adherent cells were further incubated for 18 h in medium with or without *E. coli* LPS (10 ng/ml, Sigma), LAM (10 μg/ml; gift from G. Puzo, IPBS, Toulouse, France), anti-DC-SIGN antibodies (50 μg/ml, clones 1B10 and 120507), and isotype control (50 μg/ml). IL-10 in culture supernatant was then measured by ELISA (high-sensitivity kit, R&D Systems).

### Immunohistochemistry

Lung biopsies were stained for DC-SIGN and *M. tuberculosis* using a DC-SIGN antibody (clone 1B10) and a homemade anti-*M. bovis* BCG polyclonal rabbit serum, respectively, as previously described [19].

### Confocal Microscopy

Mφs were allowed to adhere for 15 min on polylysine-coated cover slips before fixation. Cells were then permeabilized with 0.05% saponin, immunostained, and analysed as described [19]. DC-SIGN was detected using clone 120507 mAb and a Cy3-conjugated secondary mAb (Amersham, Little Chalfont, United Kingdom). Examination was realized using a confocal microscope (Zeiss, Oberkochen, Germany) and the software LSM 510 v3.2 (Zeiss).

### RT-PCR

Cells were recovered and homogeneized in Trizol (GIBCO BRL/Invitrogen, Carlsbad, California, United States). Total RNAs were extracted by classical chloroform procedure. Purified RNAs (100 ng) were used to amplify the DC-SIGN and GAPDH (glyceraldehyde-3-phosphate dehydrogenase) transcripts using the Onestep RT-PCR kit (Qiagen, Valencia, California, United States). Oligonucelotides hCD209F (5′- ATTTTC CAACTCATTTTCAGCC-3′) and hCD209R (5′- TCTCACAGAAAGAGGAGGACAC-3′) were used to amplify a 193-base pair product in the exon 7 of DC-SIGN. Oligonucelotides GAPDHRT1 (5′- GAGTCAACGGATTTGGTCGTAT-3′) and GAPDHRT2 (5′- AGTCTTCTGGGTGGCAGTGAT-3′) were used to amplify a 542-base pair product from exons 2 to 8 of GAPDH. Reverse transcription was conducted for 30 min at 50 °C. PCR consisted of 15-min activation (95 °C), and 35 cycles of 1-min denaturation (94 °C), 1-min annealing (55 °C), and 1-min elongation (72 °C). RT-PCR products were analyzed on a 1% agarose gel.

## Results

### Alveolar CD11b-Expressing Cells Over-Express DC-SIGN in Patients with TB

Given the important role of DC-SIGN in mycobacterial interactions with human MoDCs in vitro [7–9], we asked whether the lectin might interact with the tubercle bacillus during the natural course of TB in vivo in the lungs. Flow cytometry analysis of BAL cells from TB patients typically showed two major cell populations (Figure 1A, upper left panel). One of them, noted as R1, essentially consisted of CD3^+^ and either CD4^+^ or CD4^−^ T lymphocytes (Figure 1A, upper right panel). Cells from the other major population, noted as R2, were all CD3^−^ (data not shown). Total cells were co-stained with antibodies directed against CD11b (type 3 complement receptor [CR3] α chain) and DC-SIGN. Cells from R2 were all CD11b^+^ (Figure 1A, middle left panel). In patients with TB, a variable proportion of this cell population was DC-SIGN^+^ (27% in the patient shown in Figure 1A, middle right panel). By contrast, R2 cells from patients without TB were nearly all CD11b^+^DC-SIGN^−^ (results obtained with BAL cells from a patient with sarcoidosis are shown in Figure 1A, lower left and right panels). Results obtained for all individuals included in the study are shown in Figure 1B. The proportion of CD11b^+^DC-SIGN^+^ cells in BALs was not correlated with gender (19.8 ± 20.0% in males vs. 16.3 ± 13.7% in females; Mann-Whitney test *p* = 0.786), and very weakly with age, as assessed by Spearman correlation coefficient (*ρ*_s_^2^ = 0.094, *p* = 0.0078), which most likely reflected the bias in population sizes depending on age and pathology. Indeed, 72% of the whole population was aged ≤15 y and included 85% of the total TB cases. When the whole population was divided in individuals of ≤15 or ≥20 y of age, correlation with age was no longer observed (*ρ*_s_^2^ = 0.003, *p* = 0.813, and *ρ*_s_^2^ = 0.0002, *p* = 0.777, respectively). By contrast, the proportion of CD11b^+^DC-SIGN^+^ cells in BALs was highly related to the pathology. Indeed in patients with TB, 11%–71% of CD11b^+^ cells (31.2 ± 13.4%) were found to express the lectin. By contrast, CD11b^+^ cells from patients with other lung diseases or from control individuals barely expressed DC-SIGN (2.9 ± 1.8% in patients with asthma, Mann-Whitney test *p* < 10^−7^; 3.7 ± 4.1% in patients with sarcoidosis, Mann-Whitney test *p* < 10^−6^; 1.9 ± 2.0% in other individuals, Mann-Whitney test *p* < 10^−5^; all as compared to the group of patients with TB). Immunohistochemistry analysis of lung biopsy samples from patients with TB (*n* = 4) revealed that DC-SIGN^+^ cells localized mostly in cellular infiltrates outside granulomas and were tightly associated with bacilli (representative result from one patient is shown in Figure 1C). By contrast, and in accordance with results from flow cytometry, very few DC-SIGN–expressing cells were detected inside and outside granulomas in lung biopsy samples from patients with sarcoidosis (*n* = 3; representative result from one patient is shown in Figure 1D).

**Figure 1:**
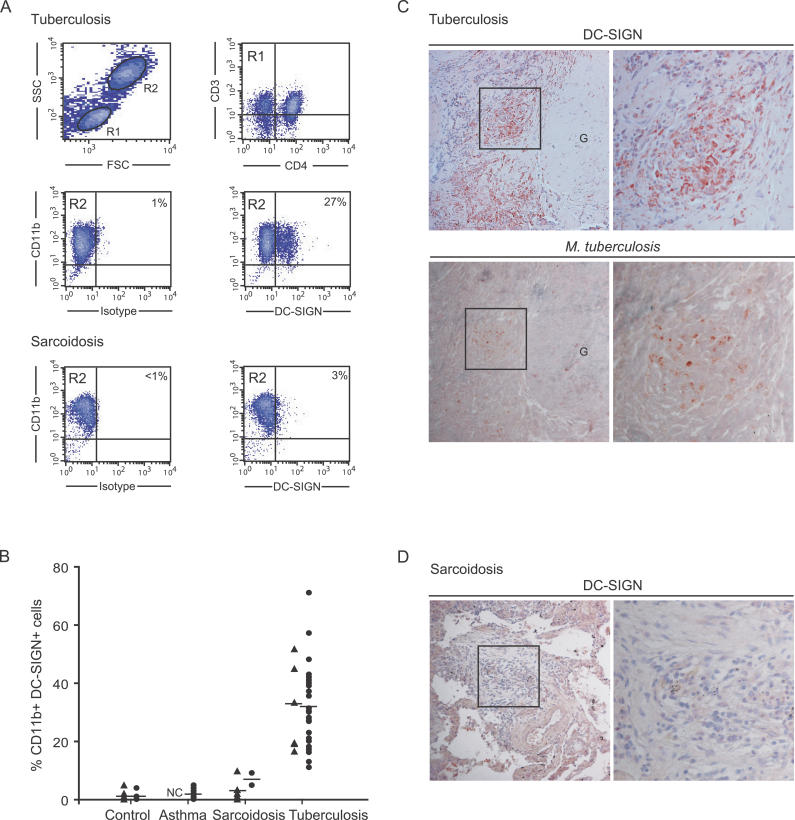
Alveolar CD11b^+^ Cells Over-Express DC-SIGN in Patients with TB (A) BAL cells from a patient with TB (upper four panels) and from a patient with sarcoidosis (lower two panels) were analyzed by flow cytometry. Expression of CD3 and CD4 was analyzed on cells from R1. CD11b and DC-SIGN expression was analyzed on cells from R2. (B) Distribution of the proportion of CD11b^+^DC-SIGN^+^ cells in BALs according to pathology and age. Black circles indicate ≤15 y of age; black triangles indicate ≥20 y; NC, no case. (C) DC-SIGN (upper panels) and *M. tuberculosis* (lower panels) immunodetection in serial sections of a lung biopsy from a patient with TB. The pictures are representative of results obtained with samples from a total of four patients. G, granuloma. (D) DC-SIGN immunodetection in a lung biopsy from a patient with sarcoidosis. The pictures are representative of results obtained with samples from a total of three patients. In (C) and (D), magnification in left panels is 100×, and regions in squares are shown at higher magnification in right panels.

### Alveolar DC-SIGN–Expressing Cells in Patients with TB Are Mφs

The ability of CD11b^+^DC-SIGN^+^ BAL cells to bind to the plastic (Figure 2A), together with their morphology (Figure 2B), strongly suggested a Mφ phenotype. In order to precisely define this phenotype, BAL cells from patients with TB were stained for DC-SIGN and for a number of markers specific to various DC subtypes, monocytes, and Mφs, and were analyzed by flow cytometry (Figure 2C and 2D). Four DC subtypes have been described in the human lungs [21], namely myeloid DCs type 1 (MDC1) expressing BDCA-1 and HLA-DR, myeloid DCs type 2 (MDC2) which are BDCA-3^hi^HLA-DR^+^CD32^−^CD64^−^, plasmacytoid DCs (pDCs) expressing BDCA-2 and CD123, and CD1a^+^ Langerhans cell type DCs. As shown in Figure 2C and 2D, DC-SIGN–expressing cells were clearly negative for BDCA-1 and BDCA-2. Cells were also BDCA-3^lo^CD32^+^CD64^+^, and they were negative for the Langerhans cell markers CD1a and CD207 (langerin; data not shown). Finally the cells did not express the DC maturation marker CD83. It is thus very unlikely that these cells constitute either myeloid or plasmacytoid DCs [21]. Cells were also negative for the monocyte marker CD14 (Figure 2C). However, the cells expressed the myeloid marker CD68 (Figure 2C). Additional staining helped to further define the phenotype of these cells. Indeed DC-SIGN^+^ cells in R2 (see Figure 1A) clearly expressed the co-stimulation and presentation molecules CD40, CD86 (B7–2), and HLA-DR, as well as the antigen uptake molecules CD11b, CD11c (CR4 α chain), CD206 (mannose receptor), CD16 (FcγRIII), CD32 (FcγRII), CD64 (FcγRI), and the Toll-like receptors (TLRs) TLR2, TLR4, and TLR9. Altogether, these results indicate that the CD11b^+^DC-SIGN^+^ cells observed in BALs from patients with TB most likely constitute alveolar Mφs. Furthermore, DC-SIGN^+^ and DC-SIGN^−^ CD11b^+^ alveolar Mφs were found to express HLA-DR and CD86 at similar levels (data not shown), indicating that DC-SIGN expression by these cells did likely not result from cell activation.

**Figure 2:**
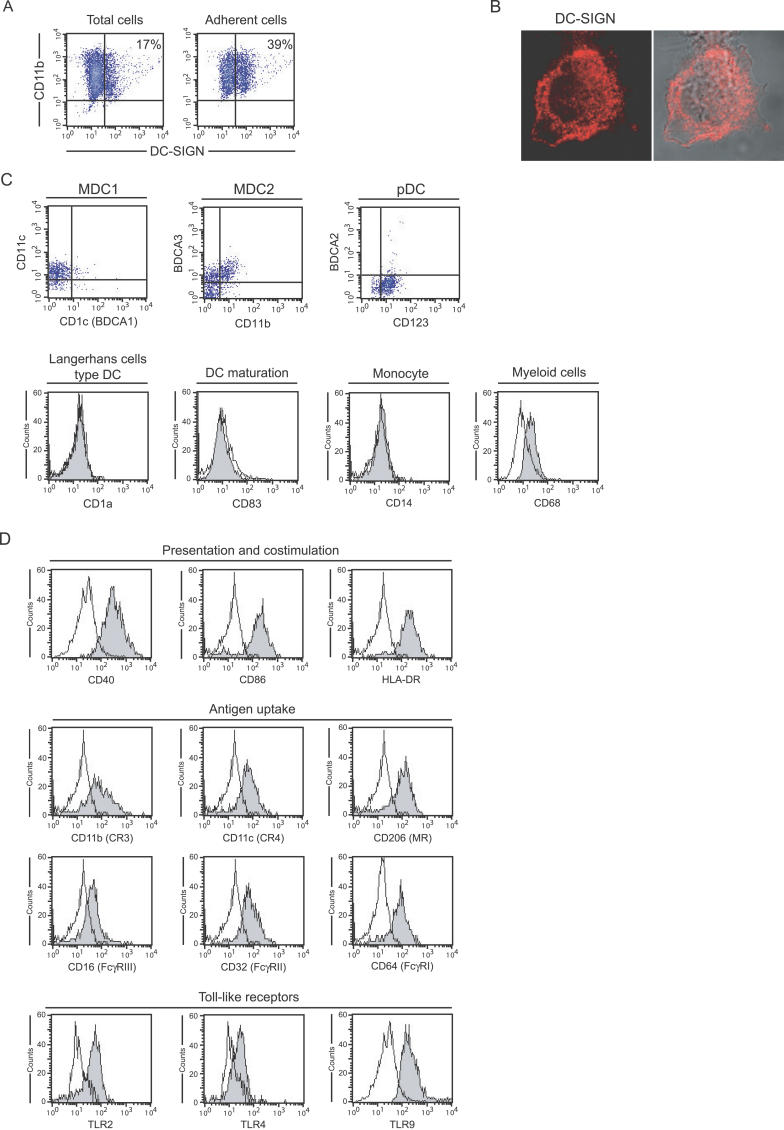
Alveolar DC-SIGN^+^ Cells in Patients with TB Are Mφs (A) Total BAL cells from a patient with TB were allowed to adhere to the plastic for 1 h at 37 °C in complete medium. CD11b and DC-SIGN expression was analyzed by flow cytometry before (left) and after (right) adherence. (B) Surface and intracellular DC-SIGN (red) expression by an adherent alveolar cell examined under the confocal microscope. (C) Flow cytometry analysis of surface expression of BDCA-1 (CD1c), BDCA-2, BDAC-3, CD1a, CD11b, CD11c, CD14, CD68, CD83, and CD123 in DC-SIGN^+^ BAL cells from a patient with TB. (D) Flow cytometry analysis of surface expression of CD40, CD86, HLA-DR, CD11b, CD11c, CD206, CD16, CD32, CD40, CD64, TLR2, TLR4, and TLR9 in DC-SIGN^+^ BAL cells from a patient with TB. In (C) and (D), analysis was performed on DC-SIGN–expressing cells in R2, as shown in Figure 1.

### DC-SIGN Is Induced on Resident Alveolar Mφs upon *M. tuberculosis* Infection

We next sought to address whether the DC-SIGN^+^ Mφs detected in BALs from patients with TB were resident alveolar Mφs or were derived from DC-SIGN^+^ monocytes recruited from the circulation during infection. Peripheral blood mononuclear cells (PBMCs) from patients with TB (*n* = 3) were analysed for DC-SIGN expression by flow cytometry. DC-SIGN could be detected neither in these cells nor in PBMCs from healthy blood donors (results from one representative experiment is shown in Figure 3A). To assess the induction hypothesis, DC-SIGN^−^ alveolar Mφs were purified by adherence from patients with asthma (*n* = 3). Cells were then infected with *M. tuberculosis* at a MOI of 1 bacterium/cell, or treated with IL-4, TNF-α, or *E. coli*–derived LPS. IL-4 was used as a control because this cytokine has been shown to induce DC-SIGN expression in human monocytes [22]. TNF-α was used to assess whether an inflammatory context, like the one encountered in lungs from patients with TB, could induce DC-SIGN. Finally, LPS was included in order to assess whether TLR ligation may result in DC-SIGN induction, as recently reported in human circulating blood monocytes [23]. Flow cytometry analysis after 48 h showed a marked expression of DC-SIGN on the surface of the cells, both when infected with *M. tuberculosis* and when treated with IL-4, as compared to untreated cells (Figure 3B). By contrast, neither TNF-α- nor LPS-treated cells were found to express the lectin. Interestingly, in cells infected with a GFP-expressing strain of *M. tuberculosis,* both GFP^+^ (infected) and GFP^−^ (uninfected) cells were found DC-SIGN^+^, indicating that DC-SIGN induction likely relies on a soluble factor secreted by the host and/or the pathogen and that has yet to be identified. RT-PCR analysis of the DC-SIGN transcript at various times post-infection (Figure 3C) further confirmed that *M. tuberculosis* infection induces DC-SIGN expression in resident alveolar Mφs as soon as 1 h after infection. Because IL-4 and IL-13 were previously shown to induce DC-SIGN expression in monocytes [22], we wished to assess whether these cytokines might account for DC-SIGN expression by alveolar Mφs in vivo in the lungs of patients with TB. We collected BAL fluids from a number of patients with TB or with other pathologies, and we measured IL-4 and IL-13 amounts by ELISA. IL-4 was detected in some samples, but without any apparent correlation with either the pathology or the amount of DC-SIGN-expressing alveolar Mφs in the corresponding samples (Table 1). IL-13 was not detectable in all samples tested (data not shown). This indicates that IL-4 and IL-13 are likely not responsible for DC-SIGN induction in vivo in the lungs of *M. tuberculosis*–infected patients.

**Figure 3:**
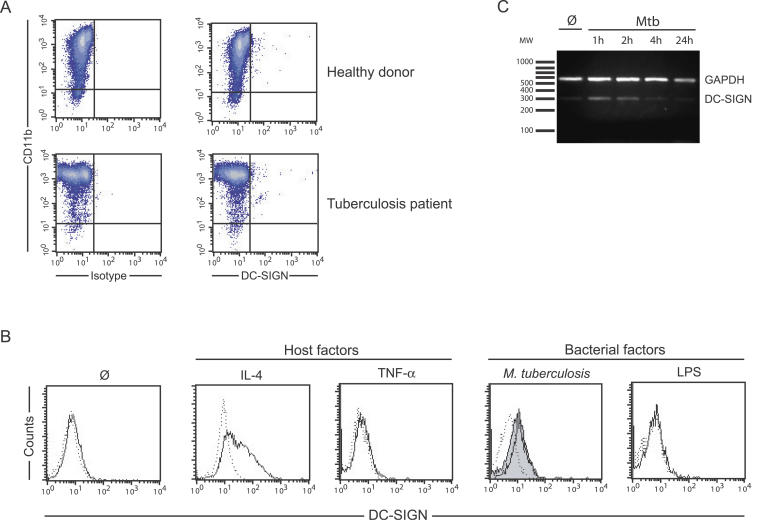
DC-SIGN Is Induced on Resident Alveolar Mφs upon *M. tuberculosis* Infection (A) Flow cytometry analysis of CD11b and DC-SIGN expression by PBMCs from a healthy donor (upper panels) and a patient with TB (lower panels). (B) Adherent DC-SIGN^−^ alveolar Mφs from a non-tuberculous patient were infected with a GFP-expressing strain of *M. tuberculosis* at a MOI of one bacterium per cell, or treated with IL-4, TNF-α, or LPS, or left untreated (ø control). After 48 h at 37 °C, cells were recovered and DC-SIGN expression was analyzed by flow cytometry. In *M. tuberculosis* panel, the grey area corresponds to GFP^+^ (infected) cells, and the plain line corresponds to GFP^−^ (uninfected) cells. (C) Cells infected with *M. tuberculosis* at a MOI of 1 for 1, 2, 4, or 24 h were analysed by RT-PCR for DC-SIGN and GAPDH mRNAs.

**Table 1:**
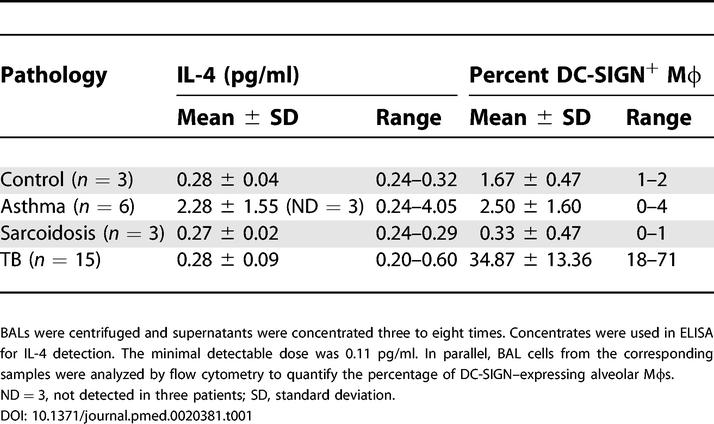
IL-4 in BAL Fluids Does Not Correlate with Pathology nor with Amount of DC-SIGN-Expressing Alveolar Mφs

### DC-SIGN Mediates *M. tuberculosis* Binding to Alveolar Mφs from Patients with TB

We next wished to evaluate the possible functional consequences of DC-SIGN induction in alveolar Mφs in patients with TB. DC-SIGN has previously been shown to be a major *M. tuberculosis* receptor on human MoDCs [7]. We thus evaluated lectin's contribution to mycobacterial binding to and entry into DC-SIGN^+^ alveolar Mφs. Total BAL cells from two patients with TB were incubated with a GFP-expressing strain of *M. tuberculosis* for 4 h at 4 °C in the presence or absence of blocking anti-CD11b (CR3) or anti–DC-SIGN or control isotype antibodies. The cells were stained for CD11b and DC-SIGN using phycoerythrin- or allophycocyanin-conjugated antibodies. Flow cytometry analysis revealed that among CD11b^+^ alveolar Mφs, DC-SIGN–expressing cells were much more prone to infection than their DC-SIGN^−^ counterparts. After infection, almost 80% of CD11b^+^DC-SIGN^+^ versus only 20% of CD11b^+^DC-SIGN^−^ cells were found GFP^+^ (Figure 4A and 4B). Accordingly, anti-DC-SIGN antibodies could inhibit >60% of *M. tuberculosis* binding to DC-SIGN–expressing Mφs (Figure 4A and 4B). The antibodies had a slight positive effect on mycobacterial binding to CD11b^+^DC-SIGN^−^ cells, which was likely due to more mycobacteria available for binding to these cells in the presence of the antibody (Figure 4A and 4B). Conversely, anti-CD11b antibodies could inhibit *M. tuberculosis* binding to DC-SIGN^−^ alveolar Mφs of almost 50% in average, whereas they had only a very minor effect on binding to CD11b^+^DC-SIGN^+^ cells (Figure 4A and 4B). In order to confirm that DC-SIGN constitutes a major *M. tuberculosis* receptor in the context of a Mφ cell and in the presence of other mycobacterial receptors, THP1 human Mφs were transduced with a lentivirus-based DC-SIGN-encoding vector and were used in *M. tuberculosis*–binding experiments. *M. tuberculosis* was found to bind to DC-SIGN–expressing THP1 Mφs by greater than 10-fold more than to THP1 cells (Figure 4C). Anti-DC-SIGN antibodies could fully inhibit mycobacterial binding to DC-SIGN-expressing cells, whereas they had virtually no effect on binding to THP1 cells. Altogether, these results indicate that DC-SIGN expression renders Mφs, and in particular alveolar Mφs in the lungs of patients with TB, more susceptible to infection than their DC-SIGN^−^ counterparts. Confocal microscopy examination of alveolar Mφs infected with GFP-expressing *M. tuberculosis* and subsequently immunostained for DC-SIGN, showed, as in MoDCs [7], a marked recruitment of the lectin at the site of bacterial attachment and in the nascent phagosome (Figure 4D, upper and middle panels), followed by exclusion of the receptor from the mycobacterial vacuole once the bacillus was engulfed, most likely as a result of receptor recycling (Figure 4D, lower panels).

**Figure 4:**
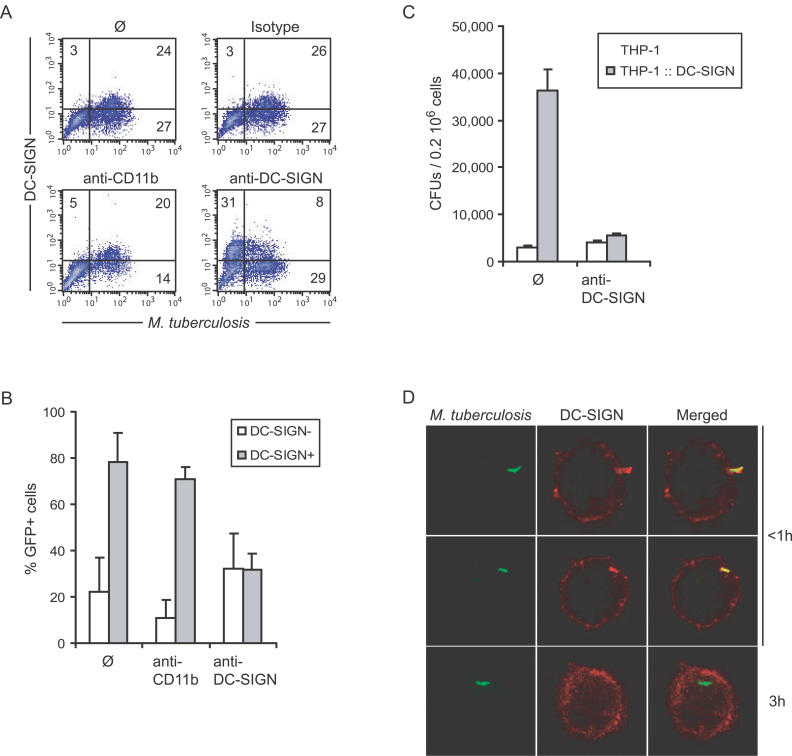
DC-SIGN Mediates *M. tuberculosis* Binding to Alveolar Mφs from Patients with TB (A) Alveolar Mφs from a patient with TB were infected with GFP-expressing *M. tuberculosis,* in the absence (ø; upper left panel) or the presence of control isotype (upper right panel), anti-CD11b (lower left panel), or -DC-SIGN (lower right panel) blocking antibodies. In the upper panels, cells were then stained with fluorescent PE-conjugated anti-DC-SIGN and APC-conjugated anti-CD11b antibodies. In lower panels, fluorescent antibodies were added together with blocking antibodies (same clones). (B) Proportion of GFP^+^ cells in DC-SIGN^−^ (open bars) and DC-SIGN^+^ (grey bars) alveolar Mφs as calculated from (A) using BALs from two patients with TB. THP1 Mφs expressing or not expressing DC-SIGN (THP1::DC-SIGN) were used in a binding experiment with *M. tuberculosis* H37Rv, in the presence or absence of anti-DC-SIGN antibodies. (D) Confocal microscopy examination of adherent DC-SIGN^+^ cells infected with GFP-expressing *M. tuberculosis* for various times.

### DC-SIGN Does Not Mediate IL-10 Secretion in Alveolar Mφs

Besides its function in pathogen recognition and phagocytosis, DC-SIGN has been proposed to play a role in triggering intracellular signals and cytokine secretion. In particular, it has previously been reported that DC-SIGN ligation by LAM synergizes with TLR4 ligation by LPS to potentiate IL-10 secretion by MoDCs [8]. We sought to determine whether this might also be the case in alveolar Mφs, which also express TLR4 (see Figure 2C). Alveolar Mφs from three patients with TB were purified by adherence and stimulated for 18 h with LPS alone or LPS and *M. tuberculosis*–derived LAM, in the presence or absence of anti-DC-SIGN or control isotype antibodies. Although basal IL-10 production differed among Mφ preparations, treatment of the cells with LPS resulted in a slight increase in IL-10 secretion in all cases, as revealed by ELISA (Table 2). Treatment of the cells with LAM alone had virtually no effect on IL-10 production. By contrast with what has been reported in MoDCs [8], LAM did clearly not synergize with LPS to induce production of the cytokine by alveolar Mφs (Table 2). This was confirmed by measuring IL-10 in BAL fluids from patients with TB or with other pathologies (Table 3). IL-10 was detected in some samples but not systematically in samples from patients with TB that contained DC-SIGN-expressing Mφs.

**Table 2:**
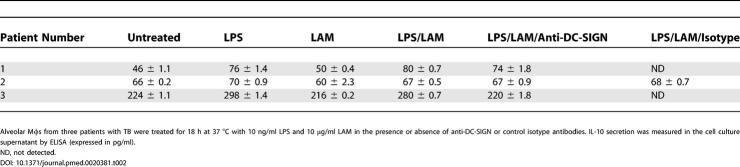
DC-SIGN Ligation by LAM Does Not Potentiate IL-10 (in pg/ml) Production by LPS-Stimulated Alveolar Mφs

**Table 3:**
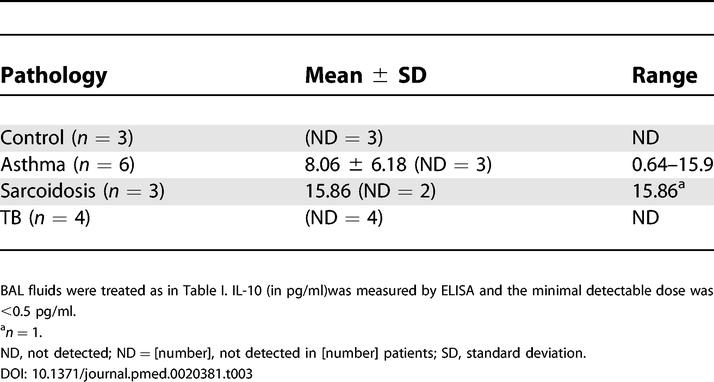
IL-10 Is Not Detected in BAL Fluids from Patients with TB

## Discussion

Our study reveals the presence of a novel DC-SIGN–expressing subset of alveolar Mφs in the lungs of patients with TB. In patients with TB, this Mφ subset represents up to 70% of total CD11b^+^ Mφs. By contrast, in other patients with non-tuberculous lung diseases, as well as in control individuals, DC-SIGN Mφs represent only 3% on average of CD11b^+^ Mφs. Our results strongly suggest that DC-SIGN^+^ Mφs arise from induction of the *DC-SIGN* gene during infection. IL-4 and IL-13, two cytokines known to induce DC-SIGN expression in monocytes [22], are unlikely to account for DC-SIGN expression by alveolar Mφs in patients with TB, because these cytokines were either not detected (IL-13) or detected in variable amounts and independently of pathology (IL-4) in BAL fluids from a number of different patients. Recently, DC-SIGN induction has been demonstrated in human circulating blood monocytes treated with TLR agonists [23]. This is unlikely to be the case here because we have shown that LPS treatment of DC-SIGN^−^ alveolar Mφs obtained from individuals without TB could not induce DC-SIGN expression in these cells (see Figure 3B). Inflammation alone is unlikely to induce DC-SIGN, because alveolar Mφs from patients with asthma or sarcoidosis were mostly DC-SIGN^−^, whereas these diseases are characterized by a marked inflammatory response in the lungs. The fact that both *M. tuberculosis*–infected and uninfected alveolar Mφs up-regulate DC-SIGN during ex vivo infection (see Figure 3B) is of interest, as it strongly suggests that soluble factor(s) from the host and/or the microbe can induce DC-SIGN in bystander DC-SIGN^−^ Mφs. These factors will have to be defined in future studies. In particular, the role of *M. tuberculosis*–derived lipids and proteins that are secreted by the bacterium prior to uptake by host cells and from inside infected cells [24,25] will have to be investigated. In addition, the possible role of host factors, such as IL-15, will have to be examined in this respect. Indeed, IL-15 has recently been shown to induce DC-SIGN expression in human monocytes in vitro [23], and alveolar Mφs from patients with TB have been reported to produce this cytokine ex vivo [26].

So far, mycobacterial receptors on Mφs have been mostly characterized using model Mφs, namely mouse bone marrow–derived or human monocyte–derived Mφs [27]. Very few reports are available on mycobacterial receptors on alveolar Mφs, and all of them used cells from healthy individuals or laboratory animals. The main mycobacterial receptors identified on human alveolar Mφs are CRs, especially CR4 and CR3 on human cells [28–30], the surfactant protein A receptor [31], and the mannose receptor [32]. Other studies in murine alveolar Mφs confirmed these findings and added other receptors, such as scavenger receptors, to the list [33]. However, to our knowledge, ours is the first study of *M. tuberculosis* receptors on alveolar Mφs in patients with TB. Our results suggest a novel scenario of alveolar Mφ infection during TB. In this scenario, CRs likely mediate most of cell infection in a naive host, and DC-SIGN–expressing Mφs become privileged target cells for the bacillus once the infection is established. Furthermore, DC-SIGN induction in bystander cells may be of advantage for the tubercle bacillus to increase its intracellular territory inside the infected host.

Apart from pathogen binding, DC-SIGN may play a role in signal transduction. In particular, DC-SIGN ligation by the mycobacterial lipoglycan LAM has been reported to potentiate TLR-4–mediated IL-10 secretion by LPS-stimulated MoDCs [8]. However, treatment of LPS-stimulated alveolar Mφs with LAM did not result in an increase of IL-10 production, which remained relatively low in both stimulated and untreated cells. Moreover, IL-10 was not detected in BAL fluids from patients with TB (Table 3). This is in accordance with previous studies reporting that IL-10 is detected in comparable amounts in BALs and lung biopsies from TB patients, and from healthy individuals [34]. IL-10 production is known to be low in Mφs as compared to in DCs. The discrepancy between our results, that show no role of DC-SIGN in IL-10 secretion, and those from Geijtenbeek et al. [8], who reported such a role, likely relies on the different cell types used in the two studies. Our results do not exclude the possibility that DC-SIGN may participate in IL-10 production by DCs in patients with TB, especially in the lymph nodes. This possibility will have to be further explored and may have important local consequences, including down-modulation of the local inflammation due to infection, either directly or by driving T lymphocytes toward a regulatory phenotype [35,36].

In conclusion, our study reveals that during the natural course of TB in human lungs, soluble host and/or mycobacterial factor(s) induce DC-SIGN expression by alveolar Mφs, which renders the cells highly prone to infection by the tubercle bacillus. DC-SIGN induction in alveolar Mφs may have important consequences on lung colonization by *M. tuberculosis,* as well as on host immune and inflammatory responses, which will require further investigation in cell and animal models, as well as in patients with TB.

**Patient Summary**

**Background**

Tuberculosis (TB) is one of the most common major infectious disease today. It is estimated that two billion people—or one-third of the world's population—are chronically infected without active symptoms. Nine million new cases of active disease are diagnosed annually, resulting in two million deaths, mostly in developing countries. TB is predominantly a lung disease. It is caused by a microbe called *Mycobacterium tuberculosis,* which infects lung cells. Patients with active disease easily infect others through coughing, sneezing, and spitting.

**Why Was This Study Done?**

Most of what we know about how *Mycobacterium* infects lung cells during the early stages of TB comes from animal studies or studies in healthy volunteers. In this study, the researchers wanted to examine whether lung cells from patients with TB were different from those of healthy people or those with different lung diseases, and what this might tell us about the way the infection spreads in the lung. In particular, they looked at the surface of the lung cells, because this is the part directly involved in the first contact with *Mycobacterium*.

**What Did the Researchers Do and Find?**

They studied 74 individuals: 40 had TB, 25 had other inflammatory lung diseases, and nine had neither active TB nor lung inflammation and served as healthy “controls.” The patients underwent a procedure (called bronchoalveolar lavage) that washes out some of the secretions and cells from the lower respiratory tract. The researchers then analysed the cells in different ways. They concentrated on a type of cell called a macrophage (the natural target of *Mycobacterium*) and found that macrophages from patients with TB had much more of a particular protein called DC-SIGN on their surface than macrophages from patients with other diseases or from the control individuals. They then took macrophages from a control individual (which thus had very low levels of DC-SIGN) and infected them with *Mycobacterium* under laboratory conditions. The researchers found that shortly after infection, not only the infected cells but also some of their neighbours started to display DC-SIGN on their surface. The researchers also found that having DC-SIGN on the surface made uninfected cells much more susceptible to infection.

**What Does This Mean?**

The results suggest that DC-SIGN has an important function in amplifying TB infection in the lung. In the long run, understanding how *Mycobacterium* infects patients and either makes them ill or establishes a chronic infection without acute symptoms should help with the development of new or better ways to prevent infection or treat disease.

**Where Can I Find More Information Online?**

The following Web sites provide information on tuberculosis.

World Health Organization pages on TB:

http://www.who.int/tb/en/

TB Vaccine Cluster page:

http://www.tb-vac.org

Tuberculosis.net, a source for TB teaching materials:

http://www.tuberculosis.net/

Wikipedia pages on TB:

http://en.wikipedia.org/wiki/Tuberculosis

MedlinePlus pages on TB:

http://www.nlm.nih.gov/medlineplus/tuberculosis.html

## Abbreviations

BAL: broncho-alveolar lavage
CR: complement receptor
DC: dendritic cell
DC-SIGN: dendritic cell–specific intercellular adhesion molecule-3 grabbing nonintegrin
ELISA: enzyme-linked immunosorbent assay
FCS: foetal calf serum
GFP: green fluorescent protein
HLA: human leukocyte antigen
IL: interleukin
LAM: lipoarabinomannan
LPS: lipopolysaccharide
mAbs: monoclonal antibodies
MoDC: monocyte-derived dendritic cell
Mφ: macrophage
PBMC: peripheral blood mononuclear cell
TB: tuberculosis
TNF: tumor necrosis factor
TLR: Toll-like receptor
